# The NLRP3 inflammasome in pathogenic particle and fibre-associated lung inflammation and diseases

**DOI:** 10.1186/s12989-016-0162-4

**Published:** 2016-09-20

**Authors:** Mutlay Sayan, Brooke T. Mossman

**Affiliations:** 1 Department of Pathology and Laboratory Medicine, University of Vermont College of Medicine, 89 Beaumont Avenue, Burlington, 05405 VT USA; 2 Department of Medicine, University of Vermont College of Medicine, 111 Colchester Avenue, Burlington, 05401 VT USA

**Keywords:** Inflammation, Fibrosis, Lung cancer, Mesothelioma, Asbestos, Silica, Airborne particulate matter, Nanotubes, Allergic airway disease

## Abstract

The concept of the inflammasome, a macromolecular complex sensing cell stress or danger signals and initiating inflammation, was first introduced approximately a decade ago. Priming and activation of these intracellular protein platforms trigger the maturation of pro-inflammatory chemokines and cytokines, most notably, interleukin-1β (IL-1β) and IL-18, to promulgate innate immune defenses. Although classically studied in models of gout, Type II diabetes, Alzheimer’s disease, and multiple sclerosis, the importance and mechanisms of action of inflammasome priming and activation have recently been elucidated in cells of the respiratory tract where they modulate the responses to a number of inhaled pathogenic particles and fibres. Most notably, inflammasome activation appears to regulate the balance between tissue repair and inflammation after inhalation of pathogenic pollutants such as asbestos, crystalline silica (CS), and airborne particulate matter (PM). Different types of fibres and particles may have distinct mechanisms of inflammasome interaction and outcome. This review summarizes the structure and function of inflammasomes, the interplay between various chemokines and cytokines and cell types of the lung and pleura after inflammasome activation, and the events leading to the development of non-malignant (allergic airway disease and chronic obstructive pulmonary disease (COPD), asbestosis, silicosis) and malignant (mesothelioma, lung cancer) diseases by pathogenic particulates. In addition, it emphasizes the importance of communication between cells of the immune system, target cells of these diseases, and components of the extracellular matrix (ECM) in regulation of inflammasome-mediated events.

## Background

Inflammation is an early response to inhaled particles in both animal models of lung injury and humans and is causally related to fibroproliferative diseases such as asbestosis and silicosis, the exacerbation of asthma and COPD by PM, lung cancers (by CS and asbestos) and mesothelioma (by certain asbestos types and erionite fibres). Historically, cells of the immune system such as macrophages, monocytes, and neutrophils have been regarded as the main players in initiating acute or chronic inflammation. However, recent studies have shown that epithelial cells of the respiratory tract and mesothelial cells lining the body cavities are capable of initiating inflammatory events after exposure to pathogenic particles in the absence of cells of the immune system via the NLRP3 inflammasome. In this review, we first describe the structure and biology of the NLRP3 (NALP3 or cryopyrin) inflammasome. We then discuss its relationship to inflammation and the development of respiratory diseases associated with inhaled pathogenic fibres (as defined as having a ≥ 3:1 length to width or aspect ratio) and particles (as defined as having a < 3:1 aspect ratio). Papers shedding light on mechanisms by which these agents cause inflammasome priming and activation are highlighted. Lastly, we provide a perspective on new insights and future questions.

Prior to writing this review, we did a number of PubMed/Medline/NCBI searches, the largest data base of greater than 26 million publications in the biomedical literature. We found 25 listed reviews on inflammasomes and lung, most on the inflammasome in lung inflammation and asthma by microbial agents and fibrotic lung diseases, and none focusing on inflammasomes and particle- or fibre-induced lung diseases. We used this data base to cite refereed reports in English since initial observations on inflammasome activation by asbestos or silica [1]. For our review, we performed two-pronged search strategies for inflammasomes and asbestos (23 citations), inflammasomes and silica (43 citations), inflammasomes and airborne particulate matter (19 citations), inflammasomes and carbon nanotubes (15 citations) and inflammasomes and diesel exhaust particles (DEP) (1 cited review). Approximately 1/3 of these citations were original reports in English that we reference. The general review, including DEP-related studies, showed that oxidants and neutrophil-derived enzymes (as opposed to inflammasome-dependent caspase-1 activity) are critical for IL- β cleavage in DEP-induced inflammation [2, 3], and questioned whether DEP activated the NLRP3 inflammasome in agreement with negative initial studies [1]. Since others [4] (Rabolli et al., Part Fibre Toxicol, in press) review the cellular effects of engineered nanomaterials on inflammasomes, this review does not focus on these agents with the exception of papers on carbon nanotubes that have been studied in comparative experiments with asbestos fibres on inflammasome activation.

## Components of inflammasomes

The human immune system consists of two distinct arms, innate and adaptive, that work closely with each other in response to harmful stress situations including inhalation of pathogenic particles and infectious agents. Immune responses begin with activation of the innate immune system which temporally precedes and instructs the adaptive immune system. The innate immune system contains multiple receptors, named pattern recognition receptors (PRRs). These receptors detect impending danger such as environmental pollutants or endogenous toxic substances and elicit protective responses to contain and eliminate harmful agents. PRRs also provide the host with resistance mechanisms to tolerate damage and facilitate repair. Five distinct classes of PRRs include toll-like receptors (TLRs), nucleotide binding and oligomerization domain (NOD)-like receptors (NLRs), retinoic acid-inducible gene-I (RIG-I)-like receptors (RLRs), absent-in-melanoma (AIM)-like receptors (ALRs) and C-type Lectins (CTLs) [5]. Among these receptors, intracellular NLRs provide cytosolic integrity by serving as critical back-up defenses while membrane-bound receptors such as TLRs survey the extracellular environment.

NLRs are composed of three functional domains which include an N-terminal protein–protein interaction domain required for signal transduction, a central NACHT (or NBD) domain for oligomerization, and a C-terminal leucine-rich repeat (LRR) for ligand recognition. The LRR can also act as a repressor of NLR signaling by masking the N-terminal domain if there is no ligand stimulation [6]. Activated NLRs induce large signaling complexes to mediate innate immune responses such as the induction of inflammation or cell death. Among these NLRs, NLRP1, NLRP3, NLRP6, NLRP7, NLRP12, NLRC4, and NAIP operate through the formation of inflammasomes that also serve as caspase-1-activating platforms controlling maturation and secretion of interleukins (IL) such as IL-1β and IL-18. Inflammasomes are formed by self-oligomerizing scaffold proteins such as NLRP3 or NLRP1 that determine the components of the inflammasomes and associated activation mechanisms [7].

**Fig. 1:**
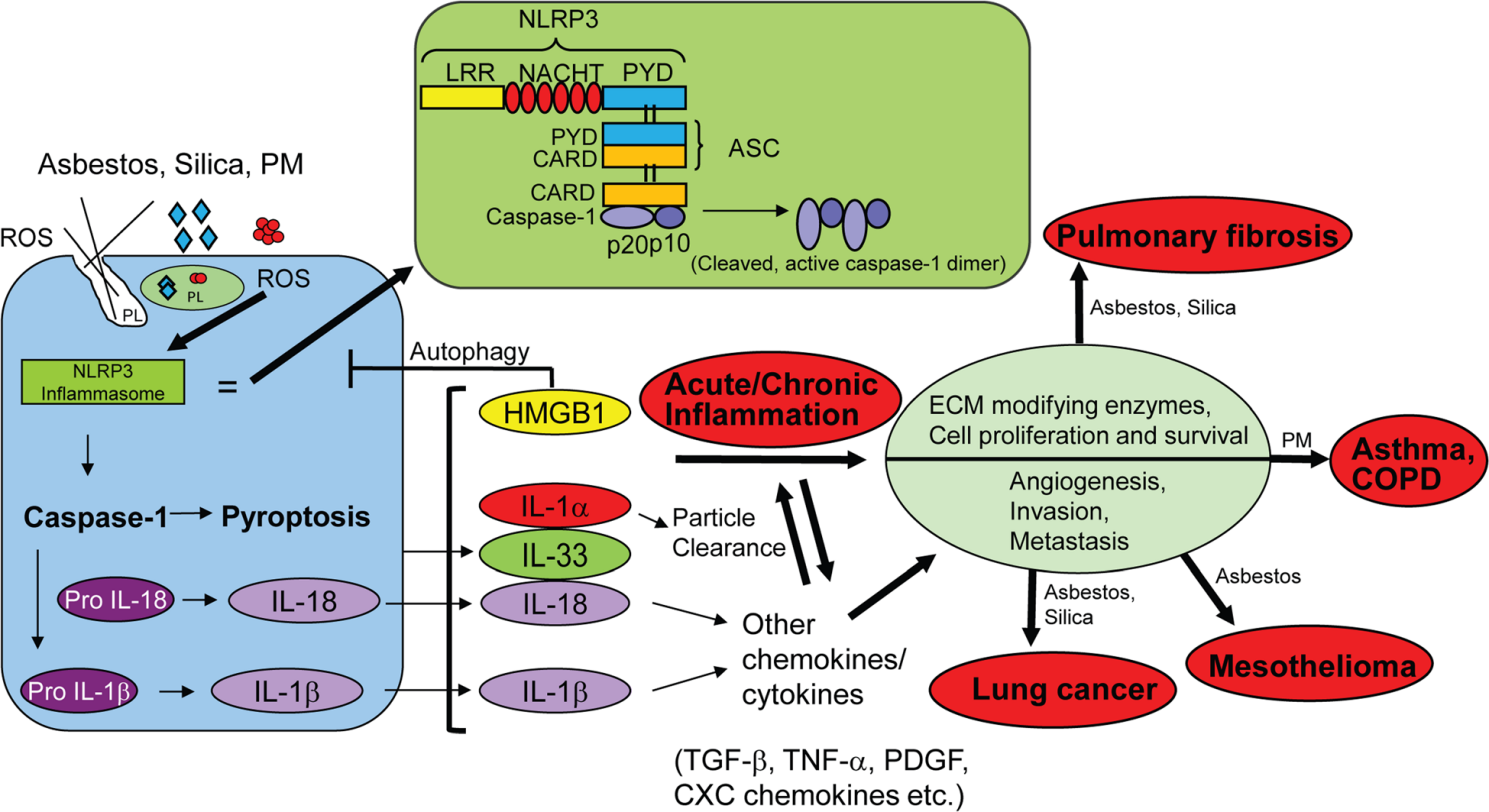
A general schematic diagram describing the components, assembly, and biologic events linked to activation of the NLRP3 inflammasome. Exposure to asbestos, erionite, CS, or PM may prime and activate the NLRP3 inflammasome via multiple mechanisms and the mechanisms detailed in Fig. 2. These fibres and particles induce dose-related damage to the cell membrane at high concentrations, are phagocytized, and can rupture phagolysosomes (PL). These processes and iron-dependent reactions on the particle surface may result in the elaboration of ROS via many routes. Inflammasome-associated caspase-1 activation leads directly to the maturation and secretion of IL-1β and IL-18. Another important function of inflammasomes is the induction of caspase-1 dependent pyroptosis which is a form of cell death characterized by both apoptosis and necrosis. This results in release of IL-1β and IL-18 as well as other inflammatory mediators such as IL-1α and HMGB1. These chemokines and cytokines either directly or indirectly lead to acute and chronic inflammation, the latter resulting in various particle and fibre-associated lung and pleural diseases. Although IL-33 is sometimes released after pathogenic particle exposures, it is unclear whether or not it plays a critical role in IL-1β maturation or production

The NLRP3 (NALP3) inflammasome is the most fully characterized inflammasome. It contains a C-terminal LRR, a central NACHT domain, and an N-terminal pyrin domain (PYD). NLRP3 binds to the adaptor protein, apoptosis speck-like protein, containing a CARD domain (ASC) which in turn recruits and activates caspase-1 (Fig. 1). Activation of NLRP3 is a multi-step process consisting of initial priming that up-regulates NLRP3 or pro-IL-1β expression levels, followed by activation signals leading to oligomerization and assembly of the inflammasome. Initially, it was thought that the priming signal depended upon Nuclear Factor-kB (NF-kB)-mediated induction of NLRP3 gene expression downstream of TLRs, NLRs and cytokine receptors. However, recent studies show that priming can also occur independently of transcriptional upregulation of inflammasome components [8]. It has also been shown that early priming does not require protein synthesis [9, 10].

**Fig. 2:**
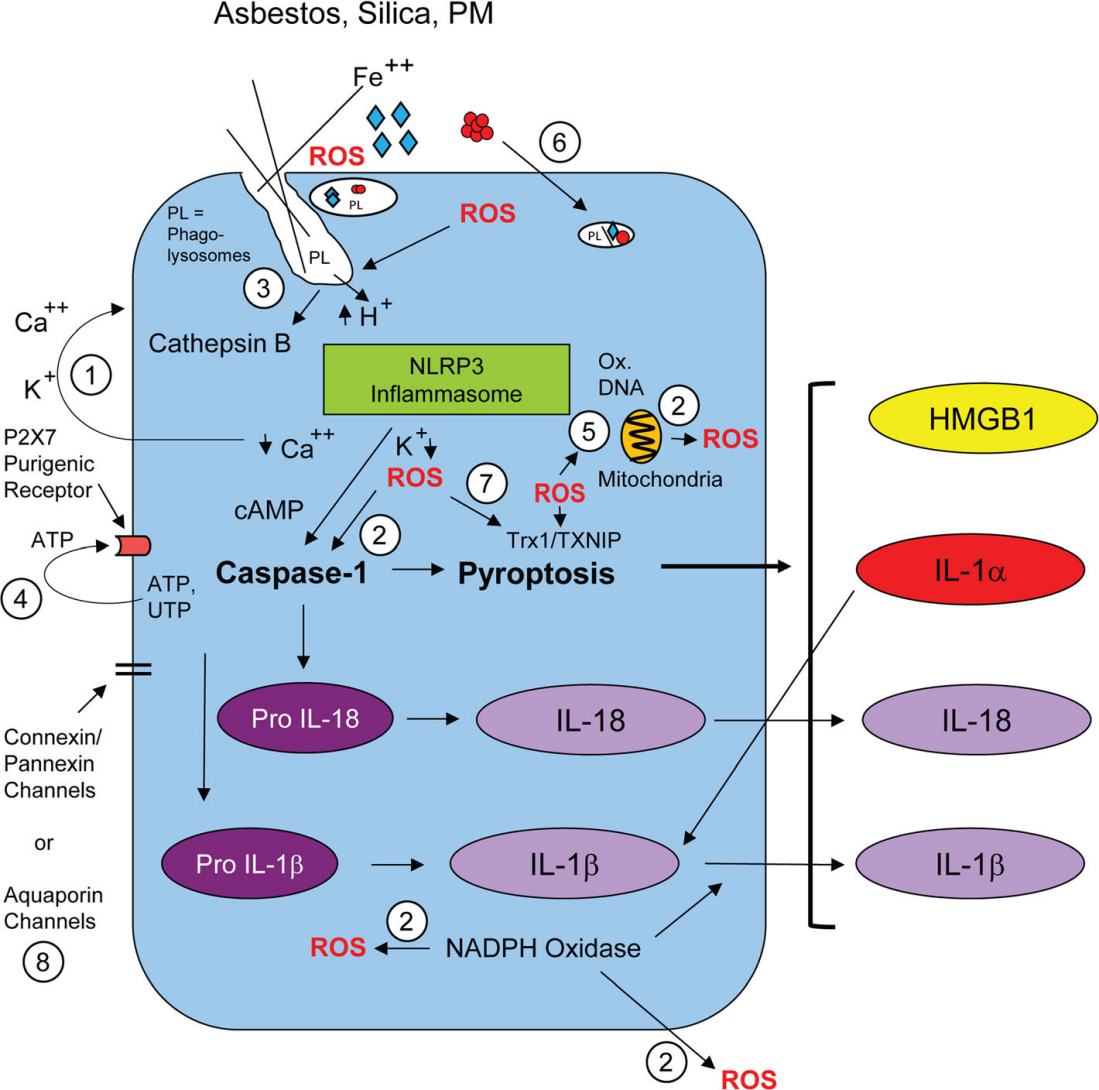
General mechanisms of inflammasome activation by pathogenic particles and fibres. Note that the complex cascades of specific proteins in these pathways are not presented. The numbers refer to individual pathways that include: 1) modifications in intracellular levels and export of K^+^ and Ca^++^; 2) formation and release of ROS both intracellularly and extracellularly that destabilize phagolysosomes, activate caspase-1; and 3) phagolysosomal disruption and increases in intracellular H^+^ (reviewed in [156, 157]). In addition to these classical pathways observed with diverse agents, studies with pathogenic particles and fibres show inflammasome priming or activation by: 4) release of ATP, ADP, and increases in purigenic receptor signaling; 5) elaboration of ROS by a multiplicity of pathways including generation mitochondrial oxidants/antioxidant enzymes and oxidation of mitochondrial DNA; 6) dose-dependent particle uptake; 7) Trx-1 oxidation and TXN1P disassociation; and 8) increases in cell volume via particle uptake, aquaporin channels (Rabolli et al., Part Fibre Toxicol, in press) and/or modulation of connexin/pannexin gap junctions that also are cytoskeletal organization proteins linked to both inflammation and immune regulation

A number of endogenous and exogenous agents induce NLRP3 activation. Currently, there is no agreement on a single mechanism by which the NLRP3 inflammasome is activated, and three different activation models have been classically recognized (Fig. 2). The first model, known as the ion flux model, suggests that changes in cytosolic levels of specific cations, such as K^+^, Ca^2+^, and H^+^, play a critical role in NLRP3 inflammasome activation [11–15]. In a second model, stimulation of the inflammasome by reactive oxygen species (ROS) occurs. Although the precise roles of ROS and steps in NLRP3 inflammasome activation remain elusive, oxidative stress has been strongly associated with NLRP3 sensing, and antioxidants attenuate caspase-1 activation [7]. Initially, mitochondrial ROS were implicated in NLRP3 activation [16]. Subsequently it was demonstrated that ROS were required for priming but unnecessary for activation [17]. In a third model of NLRP3 inflammasome activation, described as the lysosome rupture model, activation is induced by lysis of the lysosome and release of cathepsin B. These processes occur after phagocytosis of diverse particles including uric acid crystals, alum, hydroxyapatite, and amyloid β [18–21]. These mechanisms and others affecting the NLRP3 inflammasome are induced by pathogenic particles and fibres (Fig. 2).

Upon activation of NLRP3, activation of caspase-1 leads to the maturation and secretion of interleukins, IL-1β, IL-33 and IL-18 (Fig. 1). IL-1β, IL-33, IL-1α and high mobility group box 1 protein (HMGB1), as well as some heat shock proteins, facilitate many distinct systemic and localized cell to pathogenic agents, often acting as alarmins or danger signals during inflammation and other immune responses to pathogenic agents (Figs. 1 and 2). For example, IL-1β induces fever; promotes T-cell survival, B-cell proliferation, and antibody production, and mediates transmigration of leukocytes [22, 23]. Roles of IL-1β in cancer include recruitment of myeloid-derived suppressor cells (MDSCs) that impede natural killer (NK) cell development and function [24]. IL-18 can act synergistically with IL-12 to induce interferon-γ (IFNγ) production by activating T- and NK-cells [22, 23, 25]. It also down-regulates IL-22-binding protein, thereby increasing IL-22 signaling and epithelial cell repair [26]. Another important function of inflammasomes is the induction of caspase-1 dependent pyroptosis, a type of cell death characterized by both apoptotic and necrotic features [27]. This process is characterized by nuclear DNA fragmentation, plasma membrane rupture, and release of inflammatory mediators such as IL-1α, IL-1β, IL-33, and HMGB1 which play significant roles in inflammatory processes [25, 27]. In summary, multifaceted inflammasomes are critical in sensing and responding to a variety of extracellular and intracellular stresses via several pathways, and may be important in both lung defense and promulgation of chronic inflammation leading to disease.

## Relationships between inflammation and the pathogenesis of respiratory diseases

Acute inflammation is often an effective way for the body to eliminate offending pollutants and repair injury with minimal damage to host and adjacent cells; however, chronic inflammation in response to high and persistent exposures to noxious agents can cause significant cell damage and disease. Chronic inflammatory processes have also been associated with the causality of multiple malignancies and fibrosis [28]. The pro-inflammatory cytokine, IL-1β, which can be inflammasome-dependent, is a critical mediator of inflammation-promoting tumorigenesis [29–32] and fibrosis [33, 34]. Cell death mechanisms such as apoptosis and necrosis and release of chemokines/cytokines and alarmins such as HMGB1 and Tumor necrosis-alpha (TNF-α), may contribute to tumor regression, resistance to toxicity by particle and fibres, and cell growth [35, 36], by inflammasome dependent and independent pathways [37]. The transcription factors, Activator Protein-1 (AP-1) and NF-kB) that may convert inflammation-induced tumor growth to tumor regression [36], are also signals inducing the transcription or priming of pro-IL-1 β. Thus, the inflammasome and inflammation can cause both proliferation and maturation of target cells as well as immune suppression in cancers [38]. The direct role of pyroptosis, an endpoint of inflammasome activation, in causation of pro-tumorigenic or anti-tumorigenic events is still controversial, but curcumin, an anti-carcinogenic compound via its anti-inflammatory effects, causes death of mouse and human mesothelioma cells via induction of pyroptosis and activation of caspase-1, suggesting an inflammasome-dependent pathway [39].

## Pulmonary and pleural diseases associated with pathogenic inhaled particles

### Lung cancers

Increased risk of lung cancers has been associated with occupational exposures to asbestos and crystalline silica (CS), especially in smokers [40–42]. In most occupational cohorts, it has been difficult to determine the precise contributions or mechanisms of action of components of cigarette smoke and/or different types of asbestos and CS in lung cancers due to differences in particulate type, duration of exposures, dimensions, and durability in lung. However, chronic inflammation is a pathologic and diagnostic feature of lung cancers regardless of tumor subtype.

#### Inflammation in the pathogenesis of lung cancers

Alveolar macrophages (AMs) and tumor-associated macrophages (TAMs) of different subtypes are features of lung tumors. These include both M1 macrophages that stimulate the immune system, antimicrobial activity, reactive oxygen species (ROS) and reactive nitrogen species (RNS), and tumor cell killing. M2 macrophages favor immune suppression, angiogenesis, and tumor promotion. In addition, neutrophils, also components of acute and chronic inflammation, have been associated with poor outcomes in patients with lung cancers [43]. Neutrophils perpetuate inflammation due to the release of pro-inflammatory cytokines and chemokines attracting other immune cell types. Moreover, release of ROS/RNS by neutrophils and other inflammatory cell types can be mutagenic and/or promote signaling events leading to proliferation and transformation of lung cancer cells [28, 44].

In the lungs, highly inflammatory cytokines of the IL-1 family, including IL-1β, are key players mediating inflammation. For example, IL-6, a pleiotropic inflammatory cytokine often released in tandem with IL-1β, is a key growth-promoting and anti-apoptotic factor that is also involved in immune regulation and inflammation [45, 46]. IL-6 is frequently expressed in malignant respiratory epithelial cells, and high circulating levels in serum are associated with a poor prognosis in lung cancer patients [47, 48]. Its potential use as a biomarker and prognostic indicator is bolstered by the fact that it is not detected in the serum of healthy individuals and patients with benign lung diseases. In addition, growth factors that sustain proliferative signaling, survival factors that limit apoptosis, and ECM-modifying enzymes promoting angiogenesis, invasion and metastasis, provide a microenvironment favoring tumor development [43]. These factors and ROS that contribute to oncogene activation or inactivation of tumor suppressor genes, genomic instability, matrix degradation, and tumor cell proliferation are features of chronic inflammation. Also important is the upregulation of epithelial cell NADPH oxidase family proteins which generate ROS during chronic inflammation and in many cancers (Fig. 2) [49]. Another source of ROS and chronic inflammation in lung cancers includes mitochondria. Alterations in mitochondrial metabolism resulting in ROS production and oxidation of mitochondrial DNA are necessary for K-Ras-induced tumorigenicity in lung, and many signaling mechanisms coordinated at the mitochondria are being studied in the hopes of discerning new strategies for both cancer prevention and treatment [42].

#### Inflammasomes and lung cancers

Differential expression of inflammasomes have been observed in various human lung cancer lines and tissues [50]. Although inflammasome components are strikingly upregulated and mature 1 L-1β and IL-18 increased in lung cancer cell lines and tissues, variations in response occurred corresponding to histological subtype, the stage of the tumor, and its invasive potential. Highly metastatic and cisplatin-sensitive non-small cell lung cancer cells (NSCLC) expressed more inflammasome-dependent components and interleukin release than low-metastatic or cisplatin-resistant NSCLC cells, suggesting that inflammasomes are potential modulators of lung cancer development. Other studies also suggest a link between inflammasomes and lung cancers. For example, inhibition of NF-kB (a transcription factor linked to inflammasome priming) using different approaches such as RNA silencing or an IkB super-repressor, decreased both survival and proliferation of lung cancer cells [51, 52].

### Malignant mesothelioma (MM)

#### Inflammation and the pathogenesis of MM

MMs are insidious tumors that arise from mesothelial cells lining the pleural cavity in more than 90 % of cases [53]. The other 10 % of MMs originate in the peritoneum and vary rarely the pericardium and tunica of the testis and ovary. Although MMs can be idiopathic and develop after exposures to agents such as radiation, the majority (75–80 %) historically have been attributed primarily to amphibole asbestos fibres that persist in lung and pleural sites of tumor development [54]. In addition, an epidemic of MMs has been reported in certain areas of Turkey due to exposures to erionite, a needle-like non-asbestos fibres used in construction of villages [55]. MM development and manifestation of clinically detectable disease can require as long as 40 to 50 years after initial exposure to mesotheliomagenic fibres. Because diagnosis most often occurs in late-stage disease, treatment options are limited.

In contrast to shorter fibres of less than 5 μm in length, long, needle-like amphibole asbestos fibres can undergo incomplete phagocytosis by AMs or pleural macrophages and moncytes as well as mesothelial cells, events related to inflammation and inflammasome activation [1, 56] (Figs. 1 and 2). In addition to inducing acute inflammation that accompanies early injury and attempted clearance of asbestos fibres, persistent amphibole asbestos leads to the development of a chronic inflammatory response considered to play a key role in the pathogenesis of MM [57–60]. After exposure of human mesothelial cells to asbestos, chronic inflammation precedes atypical hyperplasia and the development of MM [61]. Inflammation also occurs prior to the development of MM after injection of human MM cells into immunocompromised mice, indicating that inflammation may be critical for tumorigenesis [62].

#### Inflammasomes and MM

Since asbestos and erionite fibres can prime and activate the NLRP3 inflammasome in mesothelial and epithelial cells, as well as in cells of the immune system via multiple pathways (see subsequent section on *Asbestos*), inflammasome priming and activation may play vital roles in both early lung and pleural injury as well as in inflammation, tumor initiation, and promotion. TNF-α, a protein implicated in both proliferation of mesothelial cells and prevention of asbestos-induced injury [63, 64], may also promote MM cell survival through the induction of genes encoding NF-kB-dependent anti-apoptotic molecules [35, 36] and promoting chemoresistance. TNF-α receptors are regulated by both TNF-α and IL-1α in human mesothelial cells [65] that synthesize and release both IL-1β [66] and IL-1α after injury [67, 68]. The fact that the chemotactic and autocrine growth factor [69], IL-8, is produced by human mesothelial cells in response to TNF-α and IL-1 released by macrophages or after asbestos exposure [70], supports the concept that inflammasome-mediated mature 1 L-1β release is integral to production of other cytokines and chemokines critical to the development of MMs.

### Pulmonary fibrosis

#### Inflammation and the pathogenesis of pulmonary fibrosis

Pulmonary fibrosis refers to a broad range of lung disorders characterized by the progressive and irreversible destruction of normal lung architecture, leading to scarring of the lung parenchyma. A progressive decline in lung function and impaired gas exchange due to ECM expansion leads to morbidity and mortality. The inhalation of fibres and particles such as asbestos and CS, respectively, have been associated with the development of pulmonary fibrosis, e.g., asbestosis and silicosis, in both humans and animal models [71–73].

Abnormal responses to epithelial cells that line the respiratory tract are believed to be an integral cause of scar tissue formation and the destruction of lung architecture. The recruitment of fibroblasts, proliferation of these and other mesenchymal cells that form fibroblastic foci, and release of excessive amounts of ECM components such as fibronectin, collagens, hyaluronic acid, and proteoglycans are hallmarks of the fibrotic process. A number of inflammasome-linked cytokines are associated with idiopathic pulmonary fibrosis (IPF), and fibrosis in general [74]. For example, acute pulmonary fibrotic changes associated with increased levels of IL-1β both in type II pneumocytes and AMs were observed in IPF patients undergoing acute exacerbations of disease [75]. Moreover, AMs isolated from bronchoalveolar lavage fluids (BALF) of IPF patients or individuals exposed to asbestos generated increased IL-1β mRNA levels and release of IL-1β and TNF-α [76]. IL-18 was also upregulated in the lungs of IPF patients [77].

Studies on mechanisms of silicosis and asbestosis also suggest that epithelial cell damage and persistent injury to the lung by retained durable particulates are driven by inflammatory processes [40, 42].

#### Inflammasomes and pulmonary fibrosis

CS, asbestos, and bleomycin, among other insults causing pulmonary fibrosis, activate the NLRP3 inflammasome in macrophages, monocytes and lung epithelial cells, leading to IL-1β secretion [48, 78–80]. Increased Tumor Growth Factor-β (TGF-β) production, a hallmark of inflammation and the fibrotic process, triggers the activation, proliferation, and transdifferentiation of epithelial cells and resident fibroblasts into collagen-producing myofibroblasts. This has been attributed to inflammasome-induced secretion of mature IL-1β [81]. IL-1β also stimulates the secretion of neutrophil-attracting CXC chemokines, resulting in a further influx of neutrophils that increase cell damage and oxidant release [82]. Unlike macrophages and monocytes, NADPH oxidase-derived ROS are neither required for inflammasome priming nor activation by human neutrophils, but are necessary for export of mature IL-1β from the cell [83]. IL-1β production by AMs is enhanced by fibrogenic agents that also increase production of isoforms (AA > AB > BB) of Platelet-Derived Growth Factor (PDGF), a growth-promoting cytokine and chemotactic factor [84].

A recent intriguing paper shows the importance of vimentin, a type III intermediate filament found in macrophages, fibroblasts and mesothelial cells, in regulation of the inflammasome [85]. Using three murine models (intratracheal instillation of lipopolysaccharide, bleomycin, or crocidolite asbestos at 200 μg/mouse), these investigators showed that pathophysiologic events during acute lung injury, inflammation and fibrosis were attenuated in vimentin null mice that also demonstrated decreased active caspase-1 and IL-1β levels in macrophages. Direct protein interactions between the NLRP3 inflammasome and vimentin were observed, suggesting that conformational changes of the inflammasome are important in its activation. In summary, this study supports the premise that inflammasome activation is critical to acute lung injury [86] as well as inflammatory processes leading to pulmonary fibrosis (rvewed in [33, 34]). More importantly, it points to a component of the ECM in regulation of inflammasomes.

### Asthma and chronic obstructive pulmonary diseases (COPD)

As summarized later in this review, exposure to PM can give rise to both exacerbations of asthma and COPD. These nonmalignant respiratory diseases can be debilitating and life-threatening to humans, and they can assume a number of different and overlapping pathologies and phenotypes (reviewed in [87–89]). Moreover, pulmonary hypertension is a feature of COPD which may explain the cardiovascular effects observed after exposures to PM in humans and animal models. In asthma, recruitment of neutrophils facilitates the induction of allergic sensitization and airway inflammation [90]. Other components of asthma are eosinophilic airway inflammation, airway hyperresponsiveness, and lung remodeling. COPD is a disease where a decline in lung function, increased dyspnea, and chronic sputum production (also a feature of asthma) results in deterioration of life. Like asthma, some patients with COPD have predominant eosinophilic and neutrophilic inflammation and increased chemokine production. These observations have suggested the importance of inflammation in disease causation and development. Two recent reviews detail the importance of the NLRP3 and related inflammasomes in acute lung injury and chronic inflammatory lung diseases including asthma and COPD [2, 3, 91].

## Studies on inflammasome activation by fibres and particles

### Asbestos

‘Asbestos’ is a commercial term for a group of chemically and physically distinct, naturally occuring fibres that are divided into two subgroups: needle-like amphiboles (crocidolite, amosite, tremolite, actinolite, anthophyllite), and curly serpentine (chrysotile) [53]. We first reported that crocidolite asbestos-induced (.1 and .2 mg/ml medium) release of IL-1β was dependent on the NLRP3 inflammasome in human monocyte-derived macrophages and THP-1 cells in vitro and after inhalation of asbestos (7 mg/m^3^air) by wild type and NLRP3 null mice, whereas diesel exhaust particles (.25 and .5 mg/ml medium), cigarette smoke condensate (5–10 % medium) or polystyrene beads at identical concentrations to CS and asbestos did not do so in in vitro studies [1]. Since crystalline monosodium urate (MSU) (.1 and .2 mg/ml medium) and CS (.25 and .5 mg/ml medium) exhibited similar effects to asbestos fibres, and were also phagocytized by these cell types, we proposed that cell uptake by frustrated phagocytosis was a critical mechanism of imflammasome activation by ROS production. The latter conclusion was supported by knockdown of the NADPH subunit p22^Phox^, and treatment of cells with N-acetylcysteine (NAC) or deferroxamine (an iron chelator) that inhibited mature IL-1β release. Moreover, knockdown of the antioxidant, thioredoxin-1 (Trx-1) increased amounts of IL-1β in cell supernatants [1]. Co-treatment of cells with cytochalasin β and asbestos, CS, or MSU decreased inflammasome-associated IL-1β in supernatants of cultures, confirmed the importance of phagocytosis in ROS generation in inflammasome activation (Figs. 1 and 2). Inhalation studies (7 mg/m^3^ air) showed that numbers of cells in BALF, including eosinophils and neutrophils, as well as increases in IL-1β, were significantly increased at 9 days (the time of peak inflammation in this model of murine fibrogenesis) by asbestos in wild-type mice, but significantly decreased in asbestos-exposed NLRP3 null mice.

Using siRNA approaches and chemical inhibitors, in vitro studies using crocidolite asbestos, long needle-like carbon nanotubes (CNT), short CNT and long, tangled CNT (all at 10 and 100 μg/ml medium) in human primary macrophages, showed that the high concentration of long needle-like asbestos and CNT activated the NLRP3 inflammasome despite phagocytosis of all materials that remained free in the cytoplasm. Increased enlargement and vacuolization of cells reflecting cyotoxicity, ROS production, cathepsin B activation, P2X_7_ receptor activation (a member of the family of purinoreceptors for extracellular ATP) and Src/Syk kinases were observed by asbestos fibres and long needle-like CNT, but not other materials [92]. Long needle-like CNT had more profound effects on parameters of inflammasome activation than asbestos fibres and selectively caused IL-α release. Since IL-α is released from mesothelial cells during necrosis, a cell death pathway observed in many studies using asbestos in vitro, and is considered to be an alarmin in NLRP3 inflammasome activation (Figs. 1 and 2), these important findings suggest species and cell type differences in response to asbestos fibres and CNT. Since the report by Palomaki et al. [92], a number of other investigators have confirmed that asbestos fibres and long CNT, in contrast to other shorter elongated or tangled nanomaterials or glass beads, activate the inflammasome in THP-1 human monocytes and macrophages at a range of concentrations [93–98]. These studies show that mechanisms of CNT inflammasome activation including phagocytosis, potassium efflux, cathepsin B activation, lysosomal destabilization, activation of NADPH oxidases and involvement of GTPase effector Rho-kinases (ROCK1 and 2) are similar to asbestos fibres (Fig. 2). In other studies, rare earth nanoparticles as well as long aspect ratio nanomaterials can cause inflammasome activation, IL-1β release, and autophagic flux in THP-1 cells which have a constitutively active autophagic pathway [98, 99]. Since influx of TAMs and pleural macrophages are observed in animal models of MM and human tissues, inflammasome activation in these cell types may be critical to MMs.

Other studies have focused on the mesothelial cell. For example, NLRP3 inflammasomes in human mesothelial cells in vitro are primed and activated by crocidolite asbestos (15 and 75 × 10^6^ μm/cm^2^ dish) and erionite (75 μm^2^ × 10^6^ /cm^2^ dish), not by glass beads (75 μm^2^ × 10^6^ /cm^2^ dish) [56]. It was noted that the 75 surface area dose of asbestos was toxic to cells, and erionite was non-toxic, yet dose-related increases in inflammasome priming and activation occurred. Concentration-dependent increases in NLRP3 mRNA and protein were associated with increased caspase-1 processing and activation in human mesothelial cells exposed to asbestos, and increases in secreted IL-1β and IL-18 were decreased by siRNA-induced down-regulation of NLRP3. This study also demonstrated that pretreatment of asbestos-induced mesothelial cells in vitro with anakinra, an IL-1 receptor antagonist, not only reduced IL-1β mRNA expression and protein secretion but also inhibited elevations of IL-6, IL-8, Vascular Endothelial Growth Factor (VEGF), and HMGB1 proteins, as well as NLRP3 mRNA expression (i.e., priming). These results suggest that crocidolite asbestos-induced release of IL-1β and other cytokines is predominantly regulated by the NLRP3 inflammasome.

High iron-containing amphibole asbestos fibres such as crocidolite and amosite that are associated with the development of human MMs are potent ROS producers (reviewed in [53, 100]). Toxic concentrations of crocidolite asbestos fibres (75 × 10^6^ μm^2^/cm^2^) generating ROS via surface iron or frustrated phagocytosis of longer fibres oxidize an intracellular pool of the antioxidant, Trx-1, which results in release of Thioredoxin Interacting Protein (TXNIP) and subsequent activation of inflammasomes in a human mesothelial cell line [101]. These studies suggest that oxidation of Trx-1 and disassociation of TXN1P may be a mechanism of asbestos-induced inflammasome activation (Fig. 2). Deferoxamine, an iron-chelating agent, inhibits asbestos-induced toxicity [101, 102] and IL-1β secretion [1]. Since iron chelation does not inhibit IL-1β secretion following exposure to MSU, this mechanism appears to be specific to iron-available particles and fibres [1].

Several animal experiments have also demonstrated that asbestos fibres [1, 103, 104], Libby amphibole (LA) [1, 103, 104] and multi-walled carbon nanotubes (MWCNT) [105, 106] can lead to inflammation, inflammasome activation, and IL-1β secretion. For example, mice inhaling chrysotile asbestos (7 mg/m^3^ air) for up to 40 days exhibit increased epithelial cell proliferation, collagen deposition, mRNA expression of pro-fibrotic mediators (TGF-β, collagen I and tenascin), and increased cytokine and chemokine levels including IL-1β in BAL fluids [1, 104, 107]. Mice inhaling crocidolite asbestos (7 mg/m^3^ air) also have increased numbers of inflammatory cells and levels of pro-inflammatory chemokines IL-1β [1]. Moreover, pharyngael aspiration (2.5, 10 or 40 μg/mouse) [105] or acute inhalation [106] of MWCNT (10 mg/m^3^ air) in mice yields parameters of pulmonary inflammation including neutrophils as well as IL-1β, IL-18, IL-33, and cathepsin B, and markers of lung injury (albumin and lactic dehydrogenase) in BALF. Intrapleural injection (1 × at 5 μg/mouse) of long (>5 μm) vs. short (<5 μm) amosite asbestos, silver nanowires or carbon nanotubes MWCNT results in pleural translocation and acute inflammation with fibres beyond 4 μm remaining to cause pathogenic effects in the pleura. These results correlate with the selective effects of long fibres in the causation of MMs in many animal models [108]. The role of iron in transcriptional activation of the NLRP3 inflammasome has been studied in spontaneous hypertensive rats exposed by intratracheal instillation to naturally occurring Libby amphibole (LA) and preparations loaded with iron or pre-treated with deferoxamine (all administered 1 × at 0.5 mg/rat) [1, 103, 104]. In the LA study, it was concluded that components of the NRLP3 inflammasome were transcriptionally activated during LA-induced inflammation, but both IL-1β and NF-kB were inhibited in the presence of surface-complexed iron.

The results of these animal studies have been supported in human cells using genome-wide sequencing. For example, a recent study comparing normal human pleural mesothelial cells to human peritoneal mesothelial cells with and without exposure to crocidolite asbestos (75 × 10^6^ μm^2^/cm^2^ dish) showed greater genome-wide expression of IL-6, IL-8, and the chemokines, CXCL2 and CXCL3, in response to asbestos fibres [109]. Moreover, pleural mesothelial cells showed elevations in pro-inflammatory gene expression including Granulocyte-Monocyte- Colony Stimulating Factor (GM-CSF), IL-1β, and IL-1α. In concert, results above show that inflammasome activation and cytokine/chemokine release in response to asbestos or erionite fibres are not generally species-specific, nor restricted to cells of the immune system.

### Crystalline silica (CS)

Crystalline silica is an abundant naturally occurring mineral and is commonly encountered in a number of occupations such as construction, sand blasting, mining and milling, stone masonry, and farming [40, 110]. At airborne levels higher than current occupational standards in workplace, CS has been associated with the development of silicosis, silicotuberculosis, lung cancers, and some autoimmune disorders of the lung. The mechanisms of silicosis have been recently reviewed (see section on *Pulmonary fibrosis*) and are under investigation in a number of laboratories exploring the relationship between inflammatory potential, inflammasome activation and the development of silicosis [111–124]. These studies have yielded the following mechanisms relevant to silica-induced inflammasome activation. First, AMs and epithelial cells in the small airways of rats typically phagocytize silica crystals after inhalation (7 mg/m^3^ air), and elevations in antioxidant enzymes are observed in lung tissue [125, 126]. Many in vitro studies show that CS types are phagocytized and activate the NLRP3 inflammasome through an ROS, caspase-1 dependent pathway leading to mature IL-β release at a range of non-toxic and toxic CS concentrations and time points of exposure [1, 78, 111–114, 120, 124, 127, 128]. Lethal concentrations of silica (50 μg/ml medium) also induce apoptosis in human AMs [127]. These cell death pathways lead to the release of internalized silica particles which are encountered by adjacent phagocytic cells, thus initiating a cycle of persistent inflammation and injury at high overload concentrations both in vitro and in animal models. Acute inflammation and repair may occur at lower exposures.

Silica-induced secretion of IL-1β has been demonstrated in primary human AMs, human peripheral blood mononuclear cells, murine peritoneal macrophages, and primary murine bone marrow macrophages [1, 78, 127, 128]. No or little IL-β secretion occurs in in vitro models using silica-exposed AMs exposed to the non-fibrogenic particle, TiO_2_ at the same surface area concentrations (50 /cm^2^ dish), in cells treated with caspase-1-specific inhibitors, and in macrophages or monocytes deficient in the inflammasome components, NLRP3, ASC and caspase-1, [1, 78, 128]. In these studies, inhibiting actin polymerization via cytochalasin leads to the inhibition of silica-induced activation of the NLRP3 inflammasome. These results suggest that silica-induced NLRP3 inflammasome activation is dependent on the phagocytosis of silica crystals, potentially via scavenger receptors such as MARCO and scavenger receptor-A [111–113]. AMs isolated from MARCO null mice intratracheally instilled with 25–200 μg/ml CS for 24 h have greater inflammatory responses to CS. NLRP3 inflammasome activation and release of cathepsin B from phagolysosomes, They also demonstrate increased lysosomal permeabilization, caspase-1 activation, and acid sphingomyelinase activity compared to wild-type mice [114].

In response to silica micro- or nanoparticles, IL-1α (but not HMGB1) is rapidly released from pre-existing stocks in AMs, and promotes subsequent murine lung inflammation through the stimulation of IL-1β production in mice after pharyngeal aspiration of DQ quartz (1 × at 2.5 mg/mouse [115]. Subsequent studies in this model indicated that the absence of IL-1α, but not IL-1β was associated with impaired alveolar clearance, reduced CD11 (high) phagocytic AMs and fewer granulomas accompanied by alveolar macrophages [116]. Thus, IL-1α plays a complex role in initial production of precursor IL-1β, which is released in tandem with IL-1α. Phagocytosis of silica by AMs in vitro also results in lysosomal destabilization and rupture. A myriad of proteolytic enzymes, such as cathepsin B that is associated with the rapid activation of caspase-1 are released upstream of inflammasome formation [118]. Macrophages isolated from mice deficient in cathepsin B released significantly less mature IL-1β compared to wild type controls. Thus, the NLRP3 inflammasome may detect the contents of lysosomal rupture following the phagocytosis of silica crystals.

An additional mechanism of silica-induced inflammasome activation is release of extracellular ATP which correlates with the assembly and activation of the NLRP3 inflammasome in macrophages [119], as well as secretion of mature IL-1β [118]. Extracellular ATP released via cell death, pannexin/connexin channels, and during exocytosis of particles activates purinergic receptor signaling through the P2X7 receptor that is known to mediate K^+^ efflux and ROS production [119]. Increasing the concentration of extracellular K^+^ inhibits silica-induced (50 μg/cm^2^ dish) IL-1β secretion in LPS-primed murine macrophages, indicating a negative regulatory loop via inhibition of potassium efflux [128] (Fig. 2).

Like asbestos, silica-induced inflammasome activation correlates with ROS production since silica-induced IL-1β release and caspase-1 activity decrease after knockdown of thioredoxin or components of NADPH oxidase and chemical inhibitors of a variety of oxidants in human THP1 monocytes and murine peritoneal macrophages [1, 128]. Because silica (50 μg/cm^2^ dish)-induced cytotoxicity was not affected in macrophages from NLRP3 null mice, silica-induced AM cell death does not appear to be dependent on IL-1β-dependent release from the NLRP3 inflammasome [128].

As emphasized above, silica-induced inflammasome activation has been studied primarily in phagocytic cells of the immune system. However, both transcriptional and translational upregulation of components of the NLRP3 inflammasome as well as activation of caspase-1 and maturation of pro IL-1β has been demonstrated in primary and BEAS-2β human bronchial epithelial cells exposed to cristobalite silica (75 and 150 × 10^6^ μm^2^/cm^2^ dish) [79]. These changes were accompanied by release into medium of HMGB1, and basic Fibroblast Growth Factor (bFGF) and were inhibited in cells transfected with NLRP3 siRNA. Moreover, the conditioned medium of control vector BEAS-2B lung epithelial cells caused cell proliferation of MRC-5 fetal lung fibroblasts whereas the conditioned medium of NLRP3 siRNA-transfected cells did not. These studies suggest a causal role of the inflammasome in production of HMGB1 and bFGF by lung epithelium which subsequently causes increases in lung fibroblasts, a hallmark of silicosis. Follow-up studies in rats after intratracheal instillation of DQ12 quartz or DQ12 quartz coated with polyvinylpyridine N-oxide (both at 2 mg/rat) showed that markers of inflammasome activation in lungs and BALF were attenuated by coating of the silica particles, indicating an important role of silica surface reactivity in inflammasome interactions [129].

Several other in vivo models of silica-induced lung disease have supported observations above and the importance of the inflammation in fibrosis. For example, persistently elevated IL-1β mRNA and TNF-α expression occur over time in a murine inhalation model of silicosis (>10 mg/m^3^ air) where inflammation precedes fibrosis [130]. When silica-instilled (2.5 mg) mice were co-injected with an anti-IL-1β monoclonal antibody, depletion of IL-1β in BAL fluids, inflammation, and collagen deposition in lung as well as mRNA expression levels of TGF-β, collagen I and fibronectin in BALF were reduced [131]. These studies suggest that neutralization or deficiency of IL-1β, analogous to that occurring in NLRP3 knockout mice [128], attenuates silica-induced initiation of fibrosis by inhibiting other inflammatory and fibrogenic mediators as well. The fact that inflammatory and fibrotic responses to silica are uncoupled in MyD88 null mice [121] and NMRI mice [122] after pharyngeal instillation of silica (2.5 mg/mouse), but not in a number of rat inhalation or instillation studies (reviewed in [40]), suggest important strain and species differences in both inflammatory and fibrogenic responses to various CS types.

### Airborne particulate matter (PM)

Particulate matter (PM), a component of urban air pollution, contributes to cardiorespiratory morbidity, exacerbations of asthma and COPD [132–135]. Numerous reviews exist on the clinical features and treatment of asthma and COPD, and in general, they are regarded as inflammatory lung diseases (see previous section on *Asthma and chronic obstructive pulmonary diseases (COPD)*). The inflammasome is known to play a central role in asthma and COPD as well as pulmonary inflammation in general [2, 136–139].

Deciphering the mechanisms of actions of action of PM on priming and activation of the NLRP3 inflammasome is difficult because PM is comprised or contaminated with a number of soluble and insoluble components including mold spores, endotoxin, metals, etc. Many of these agents have been linked to inflammasome activation and inflammation both individually and in combination (reviewed in [2, 3]). Other factors influencing inflammasome activation and inflammatory effects are the size of the PM (smaller particles being generally more toxic and reactive due to a larger surface-to-mass ratio) and the source. Moreover, complex combustion-derived particles, such as diesel exhaust particles (DEP), which may trigger IL-1β production by inflammasome-independent mechanisms [2, 3], and is a component of some fine and ultrafine PMs, are complicating factors in determining the properties of PM on inflammasome activation. Although not covered extensively in this review, DEP effects on inflammasome-independent pro-inflammatory responses have been recently reviewed [2, 3]. Although ROS have been implicated as mediators of DEP-induced inflammation in a murine model of allergic airway inflammation [140], and increased IL-1β levels are observed in BALF [141] from these mice, IL-1β levels are not diminished in NLRP3 or caspase-1 null mice exposed to DEP. Neutrophil-derived enzymes, as opposed to an infammasome-mediated process, are linked to IL-1β cleavage and release [141].

A few studies have explored the effects of PM preparations on the NLRP3 inflammasome. Using PM10 (EHC-93 at a concentration of 500 μg per 9.6 cm^2^ dish/ml medium) on primary human airway epithelial cells in vitro. NLRP3 inflammasome-mediated IL-1β levels were increased in medium, and both NLRP3 inflammasomes and caspase-1 were detected histochemically in epithelial cells of the human bronchus and by immunoblots in isolated cells [142]. Moreover, use of NLRP3 null and wild type mice after a 1 × nasal instillation of PM10 (200 μg/mouse) demonstrated NLRP3-dependent production of IL-1β, neutrophilia, and increased numbers of CD11c (+hi) cell numbers in lymph nodes. Employing these same models, these investigators also demonstrated that PM10 activates an NLRP3 inflammasome/IL-1 receptor axis involving IL-1β, CCL-20, and GM-CSF production that was also associated with dendritic cell activation and neutrophilia [143]. However, even though there were profound innate immune responses, the NLRP3/IL-1R axis was dispensable for PM10-facilitated allergic sensitization. Since PM10 exposure contributes to the exacerbation of asthma, but not PM10-associated allergic sensitization, these observations suggest that PM10 inhalation is associated with innate, but not adaptive immune responses. Other studies suggest that PM 2.5 (200 μg/mouse instilled 5× intranasally) also induces airway hyperresponsiveness in NC/Nga mice that have a genetically high sensitivity to mite allergens by an inflammasome-linked mechanism [144]. Injection of soluble and insoluble components of urban PM2.5 indicated that they act synergistically on these processes. Prior PM10 exposures in a human airway epithelial cell line in vitro at concentrations used previously [142, 143], increased IL-1β secretion into medium following scratch wounding and H1N1 influenza A exposure [145] and indicated synergy of PM10 with mechanical cell injury and some viral infections. Thus PM may contribute to exaggeration of immune responses by other stimuli of the inflammasome.

## New insights and future questions

At least two steps are involved in the activation of the NLRP3 inflammasome. The first is priming that leads to increased gene expression or mRNA levels of pro IL-1β and NLRP3, and the second is the assembly and activation of the inflammasome molecular complex. Although the molecular interactions are unclear, one study has shown that NLRP3 mRNA levels are increased in a dose-related fashion after addition of crocidolite asbestos to human mesothelial cells [56]. Whereas priming was transcriptionally regulated and observed at both non-toxic and toxic concentrations (15 and 75 × 10^6^ μm^2^/cm^2^), caspase-1 activity, and release of HMGB1, IL-1β, and IL-18 were only statistically significant at high, toxic amounts of fibres. Thus, pathogenic fibres can both prime and activate the NLRP3 inflammasome albeit at different concentrations. Priming may be regulated by epigenetic mechanisms as these appear to be more common than genetic mutations in mesothelioma (reviewed by [146]). Whether these trends occur in dose-response studies with other pathogenic minerals is an important question as both in vitro and in vivo studies with particulates rarely are performed at a number of concentrations of materials, and cytotoxicity (or lack of) is only occasionally reported.

Data as a whole suggest that priming and activation of inflammasomes occur in both acute and chronic inflammation by inhaled pathogenic fibres and particles. Limited dose-response studies suggest that acute inflammation resolves and may be protective toward inhaled toxins by inducing repair responses, whereas chronic inflammation and disease are observed at high cytotoxic or overload concentrations of particles and fibres. Although not reviewed here, many studies with synthetic nanoparticles may induce priming or activation of the inflammasome at a range of concentrations reported. With use of pathogenic minerals, these protracted responses are enhanced by particulates that are durable and persist in lung (reviewed in [147]) and pleura [108, 148, 149]. Although the inflammasome has been studied most exclusively in cells of the immune system and has recently been demonstrated to be a transcriptional regulator and transcription factor in T_H_2 differentiation [150], studies using particles (silica) and fibres (asbestos) demonstrate that inflammasome activation also determines chemokine and cytokine profiles in epithelial cells and mesothelial cells in the absence of cells of the immune system. Whether NLRP3 is a transcription or epigenetic factor that affects differentiation and function of epithelial and mesothelial cells to more abnormal cell types culminating in malignant or nonmalignant lung diseases is an unexplored area.

The interplay between inflammasome activation and alarmins is complex. Alarmins may activate the inflammasome as upstream agents, or be produced as a result of inflammasome activation, i.e., HMGB1 [56]. The fact that IL-1α promotes IL-1B release in response to silica [115] also suggests that cross-talk between and co-regulation of alarmins may occur in response to particle contact or uptake. Moreover, negative feedback loops have been demonstrated with use of the IL-1R receptor blocker, anakinra [56, 151]. In the latter studies, mice exposed to crocidolite asbestos in the presence of anakinra in an accelerated genetic model of MM showed marked delay in the median time of MM onset compared to mice receiving vehicle control. Moreover, mice deficient in an important component of inflammasome assembly (Fig. 1), i.e., *Asc-*knockout mice, exhibited significantly delayed MM onset and reduced MM incidence in comparison to wild-type mice. These approaches need to be further validated in models of lung cancer and pulmonary fibrosis to allow novel therapeutic approaches to these diseases using RNA silencing or small molecule inhibitors of inflammasome pathways.

## Conclusions

The references cited in this review demonstrate that the development of lung cancers, MMs, asbestosis and silicosis, asthma, and COPD are associated with acute or chronic inflammation and activation of the NLRP3 inflammasome by several different pathogenic particles and fibres. In addition, exacerbations of COPD and allergic airway respiratory diseases, including asthma, are fueled by inflammasome-mediated chemokines and cytokines during PM exposures. A number of common cell signals contribute to NLRP3 inflammasome activation by noxious particulates as shown in Fig. 2. Other mechanisms of priming and activation of the NLRP3 inflammasome may be unique to distinct particulates. IL-1 receptor antagonists and small molecule inhibitors of the NLRP3 inflammasome have been used successfully in pre-clinical [152, 153] and clinical studies [152, 153]. In addition to these agents, inhibition of key steps in inflammasome activation may be beneficial in prevention and therapy of both non-malignant [34, 91, 154] and malignant [39, 155] diseases in the lung and pleura. The complex nature of the NLRP3 inflammasome is being unraveled, and numerous priming and activation signaling events are under intense investigation by a number of laboratories. Results should further both preventive and therapeutic approaches to diseases in the clinic.

## Abbreviations

AIM: Absent-in-melanoma
ALRs: AIM-like receptors
AMs: Alveolar macrophages
AP-1: Activator-protein-1
ASC: Apoptosis speck-like protein containing a CARD domain
BALF: Bronchoalveolar lavage fluid
bFGF: Basic fibroblast growth factor
CNT: Carbon nanotubes
COPD: Chronic obstructive pulmonary diseases
CS: Crystalline silica
CTLs: C-type lectins
DEP: Diesel exhaust particles
ECM: Extracellular matrix
GM-CSF: Franulocyte- monocyte colony stimulating factor
HMGB1: High mobility group box 1 protein
IFNγ: Interferon-γ
IL: Interleukins
IL-1β: Interleukin-1β
IPF: Idiopathic pulmonary fibrosis
LA: Libby amphibole
LRR: Leucine-rich repeat
MDSCs: Myeloid-derived suppressor cells
MM: Malignant mesothelioma
MSU: Crystalline monosodium urate
MWCNT: Multi-walled carbon nanotubes
NAC: N-acetylcysteine
NBD: Central NACHT domain
NF-kB: Nuclear factor kappaB
NK: Natural killer cells
NLRs: NOD-like receptors
NOD: Nucleotide binding and oligomerization domain
PDGF: Platelet-derived growth factor
PL: Phagolysosomes
PM: Particulate matter
PRRs: Pattern recognition receptors
PUMA: p53-upregulated modulator of apoptosis
PYD: N-terminal pyrin domain
RLRs: RIG-I-like receptors
RNS: Reactive nitrogen species
ROS: Reactive oxygen species
TAMs: Tumor-associated macrophages
TGF-β: Tumor growth factor-β1
TLRs: Toll-like receptors
TNF-α: Tumor necrosis factor-α
Trx-1: Thioredoxin-1
TXNIP: Thioredoxin interacting protein
VEGF: Vascular endothelial growth factor
